# First karyotype description of *Epiplatysspilargyreius* (Duméril, 1861) with comments on chromosome evolution in the genus *Epiplatys* Gill, 1862 (Nothobranchiidae)

**DOI:** 10.3897/compcytogen.19.151345

**Published:** 2025-06-19

**Authors:** Sergey A. Simanovsky, Alexandra Yu. Skuratovskaya, Margarita G. Simonian, Dmitry A. Medvedev, Fekadu Tefera, Alexander S. Golubtsov

**Affiliations:** 1 Severtsov Institute of Ecology and Evolution, Russian Academy of Sciences, Leninsky Prospect 33, Moscow, 119071, Russia Severtsov Institute of Ecology and Evolution, Russian Academy of Sciences Moscow Russia; 2 National Fishery and Aquatic Life Research Center, Ethiopian Institute of Agricultural Research, Sebeta, P.O. Box 64, Ethiopia National Fishery and Aquatic Life Research Center, Ethiopian Institute of Agricultural Research Sebeta Ethiopia

**Keywords:** Centromere repositioning, chromosome fissions, chromosome fusions, chromosome inversions

## Abstract

T﻿he African non-annual killifish genus *Epiplatys* Gill, 1862 (family N﻿othobranchiidae) comprises 36 valid species distributed in West, Central and East Africa. The available cytogenetic information for the genus indicates a wide variability in diploid chromosome number (2n) and number of chromosome arms (FN). Here, w﻿e report the karyotype of *Epiplatysspilargyreius* (Duméril, 1861), one of the two species with the lowest diploid chromosome number (2n = 34) in the genus, from the White Nile basin in Ethiopia. Male and female karyotypes contained 18 metacentric/acrocentric and 16 subtelocentric/acrocentric chromosomes. The number of chromosome arms is, respectively, FN = 52. Analysis of karyotype differentiation in the genus allowed us to suggest that the 2n reduction in *E.spilargyreius* and many other members of the genus *Epiplatys* is mainly due to Robertsonian translocations (reduction of 2n from 48 to 34 with stable NF = 48–52). We provide an up-to-date summary of cytogenetic data and a brief review of chromosome evolution in the genus.

## ﻿Introduction

The genus *Epiplatys* belongs to the family Nothobranchiidae and comprises 36 valid species of non-annual killifish endemic to Africa (Wildekamp 1996; Fricke et al. 2025; Froese and Pauly 2025). Representatives of the genus are distributed in West, Central and East Africa – from Senegal to Ethiopia – in small rivers, streams and swamps in rainforests and savannas (Wildekamp 1996; Froese and Pauly 2025). The genus *Epiplatys* is reported to have the widest distribution of all Nothobranchiidae (Collier 2015).

Cy﻿togenetic information for the genus, available for 22 of the 36 species, shows a wide variability in diploid chromosome number from 34 to 50 and the number of chromosome arms from 48 to 82 (Scheel 1966, 1972, 1975, 1990; Arai 2011; Collier et al. 2009; Collier 2015). Analysis of cytogenetic and phylogenetic data suggests that the ancestral diploid chromosome number was 2n = 48 and that karyotype differentiation in the genus occurred mainly by chromosomal fusions and pericentric inversions, and less frequently by chromosomal fissions (Co﻿llier et al. 2009; Collier 2015). In this paper, we describe the karyotype of *Epiplatysspilargyreius* (Duméril, 1861), one of the two species with the lowest 2n in the genus, from the White Nile Basin in Ethiopia (East Africa), to further our understanding of chromosome evolution in the genus. Previously, *E.spilargyreius* has been cytogenetically studied from Nigeria and the Democratic Republic of the Congo (West and Central Africa respectively), but only 2n has been reported (Scheel 1966, 1972, 1990; Collier 2015).

## Material and methods

Four individuals, two females and two males of *E.spilargyreius* (standard length SL = 21–28 mm), were obtained from an oxbow pond at the right bank of the Baro River, a tributary of the Sobat River, the White Nile system, at the Village of Baziel Kebele (08°18'51.2"N, 34°04'24.9"E), in south﻿western Ethiopia. Fish were collected by the Joint Ethio-Russian Biological Expedition (**JERBE**) with the permission of the National Fisheries and Aquatic Life Research Center under the Ethiopian Institute of Agricultural Research (**EIAR**) and the Ethiopian Ministry of Science and Technology. Three individuals, two females and one male, were successfully karyotyped.

After colchicine treatment, fish were euthanized with an overdose of tricaine methanesulfonate (MS-222), identified, measured with an accuracy of 1 mm, dissected for gonad examination and tissue sampling, and preserved in 70% ethanol. Speci﻿es identification was done based on morphological characters (Golubtsov et al. 1995). The experiments were carried out in accordance with the rules of the Severtsov Institute of Ecology and Evolution (**IEE**). Vouchers are deposited at the Severtsov Institute of Ecology and Evolution (Moscow), under provisional labels of JERBE.

Before preparation, fish were treated intraperitoneally with 0.1% colchicine (0.01 ml / 1 g of their weight) for 3–5 hours. After euthanasia, chromosome preparations were obtained from kidney tissue following Kligerman and Bloom (1977) with some modifications as described in Simanovsky et al. (2022). The chromosome spreads were stained conventionally with 4% Giemsa solution in a phosphate buffer solution at pH 6.8 for 8 min. The analysis was performed using an Axioplan 2 Imaging microscope (Carl Zeiss, Germany) equipped with a CV-M4+CL camera (JAI, Japan) and Ikaros software (MetaSystems, Germany). Final images were processed using Photoshop software (Adobe, USA). Karyotypes were arranged according to the centromere position following the nomenclature of Levan et al. (1964), but modified as metacentric/submetacentric (**m/sm**) and subtelocentric/acrocentric (**st/a**), similar to other cytogenetic studies on the members of the genus *Epiplatys* (Table 1). Chromosome pairs were arranged according to their size in each chromosome category. To determine the chromosomal arm number per karyotype (fundamental number, **FN**), metacentrics/submetacentrics were considered as biarmed, and subtelocentrics/acrocentrics as one arm chromosomes. The total number of complete metaphases studied for three *E.spilargyreius* individuals was 30.

**Table 1.** Cytogenetically studied taxa of the genus *Epiplatys*. Diploid chromosome number (2n), karyotypic formula, fundamental number (FN) and geographic origin. Some taxa from the original works have been renamed according to their current valid statuses (Froese, Pauly 2025; Fricke et al. 2025).

Taxon	2n	Karyotypic formula	FN	Origin	References
*Epiplatysannulatus* (Boulenger, 1915)	50	20m/sm + 30st/a	70	Guinee, Liberia	Scheel 1972, 1975, 1990; Arai 2011
*Epiplatysansorgii* (Boulenger, 1911)	46	4m/sm + 42st/a	50	Gabon	Scheel 1990; Arai 2011
	48	2m/sm + 46st/a	50	Gabon	Scheel 1990; Arai 2011
*Epiplatysbarmoiensis* Scheel, 1968	34	14m/sm + 20st/a	48	Sierra Leone, Liberia	Scheel 1972, 1990; Arai 2011
*Epiplatysbifasciatus* (Steindachner, 1881)	40	8m + 32a	48	Ghana, Nigeria	Scheel 1972, 1975, 1990; Arai 2011
*Epiplatyschaperi* (Sauvage, 1882)	50	50st/a	50	Ghana	Scheel 1972, 1975, 1990; Arai 2011
*Epiplatyschevalieri* (Pellegrin, 1904)	48	–	–	Democratic Republic of the Congo	Collier 2015
*Epiplatysdageti* Poll, 1953	50	32m/sm + 18st/a	82	Liberia	Scheel 1972, 1975, 1990; Arai 2011
	50	36m/sm + 14st/a	86	Ghana	Collier 2015
*Epiplatysduboisi* Poll, 1952	48	–	48	Congo	Scheel 1972, 1975, 1990; Arai 2011
*Epiplatysesekanus* Scheel, 1968	42	8m + 34a	50	Cameroon	Scheel 1972, 1975, 1990; Arai 2011
	42	8m/sm + 34st/a	50	Cameroon	Collier 2015
*Epiplatysfasciolatus* (Günther, 1866)	36	–	–	Liberia, Sierra Leone	Scheel 1966, 1972, 1975, 1990; Arai 2011
	38	–	–		
	40	10m/sm + 30st/a	50		
*Epiplatysgrahami* (Boulenger, 1911)	48	2sm + 46a	50	Cameroon, Equatorial Guinea	Scheel 1972, 1990; Arai 2011
*Epiplatyshuberi* (Radda et Pürzl, 1981)	46	6m/sm + 40a	52	Gabon	Scheel 1990; Arai 2011
*Epiplatysinfrafasciatus* (Günther, 1866)	48	–	–	Cameroon	Scheel 1990; Arai 2011
	48	2m/sm + 46st/a	50	Cameroon (three populations)	Collier 2015
*Epiplatyslamottei* Daget, 1954	48	4m/sm + 44a	52	Liberia	Scheel 1990; Arai 2011
	48	–	–	Guinea	Collier et al. 2009
*Epiplatysmesogramma* Huber, 1980	48	48a	48	Central African Republic	Scheel 1990; Arai 2011
*Epiplatysmultifasciatus* (Boulenger, 1913)	46	14m/sm + 32st/a	60	Zaire, Congo	Scheel 1990; Arai 2011
*Epiplatysolbrechtsi* Poll, 1941	38	12m/sm + 26st/a	50	Ivory Coast	Scheel 1990; Arai 2011
*Epiplatysroloffi* Romand, 1978	46	–	–	Liberia	Scheel 1990; Arai 2011
*Epiplatyssangmelinensis* (Ahl, 1928)	48	–	–	Cameroon	Scheel 1972, 1975, 1990; Arai 2011
*Epiplatyssexfasciatus* Gill, 1862	48	48st/a	48	Ghana, Nigeria, Cameroon, Equatorial Guinea	Scheel, 1972, 1975, 1990; Arai 2011
*Epiplatyssinga* (Boulenger, 1899)	42	–	–	Zaire	Scheel 1990; Arai 2011
*Epiplatysspilargyreius* (Duméril, 1861)	34	–	–	Nigeria	Scheel 1966, 1972, 1990; Arai 2011
	34	–	–	Democratic Republic of the Congo	Collier 2015
	34	18m/sm + 16st/a	52	Ethiopia	**This work**

## Results and discussion

**Figure 1.**
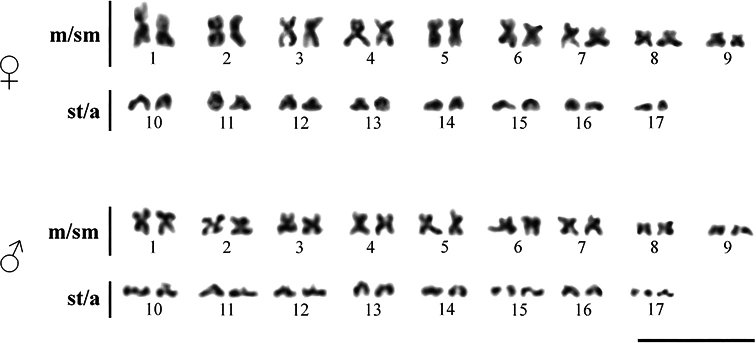
Female and male karyotypes of *Epiplatysspilargyreius* after conventional Giemsa staining. Scale bar: 10 μm.

The karyotype of the *E.spilargyreius* population from Ethiopia has 2n = 34 and consists of 18 metacentric/submetacentric and 16 subte﻿locentric/acrocentric chromosomes, FN = 52 (Fig. 1). No distinguishable sex chromosomes were observed in the female and male complements, similar to the previous cytogenetic studies on the representatives of the genus *Epiplatys* (Scheel 1966, 1972, 1975, 1990; Arai 2011; Collier et al. 2009; Collier 2015). The three studied populations of *E.spilargyreius* from Nigeria, the Democratic Republic of the Congo and Ethiopia have identical chromosome numbers (Scheel 1966, 1972, 1990; Collier 2015; this work). We could not perform a more detailed comparative analysis of karyotypes in these populations because only 2n has been reported for populations from Nigeria and the Democratic Republic of the Congo (see Table 1). Analysis of mitochondrial genes also revealed a close similarity between populations, despite the wide distribution of *E.spilargyreius* (Collier 2015). The cytogenetic characteristics (2n, FN) of the studied members of the genus *Epiplatys* are shown in Table 1.

According to phylogenetic data, the genus is divided into two major groups: “western and savanna” and “eastern and coastal” clades (Coll﻿ier et al. 2009; Collier 2015). *E.spilargyreius* belongs to the first clade, which also includes *E.bifasciatus* (2n = 40, FN = 48), *E.duboisi* (2n = 48), *E.fasciolatus* (2n = 36–40, FN = 50), *E.lamottei* (2n = 48, FN = 52) and *E.roloffi* (2n = 46) among the karyotyped species. The diploid chromosome number in this clade varies widely from 34 to 48, but FN is more stable and varies only from 48 to 52. This suggests a substantial role for structural rearrangements of centric fusion type (Robertsonian translocations) in the karyotype differentiation. It is worth noting that *E.duboisi* and *E.lamottei*, which have cytogenetic characteristics closest to the putative ancestral karyotype of the genus with 2n = 48 and FN = 48, are at the basal position on the phylogenetic tree of the clade (Collier et al. 2009; Collier 2015). Karyotypes of *E.bifasciatus*, *E.fasciolatus* and *E.spilargyreius* differ from the putative ancestral karyotype by 4, 4–6 and 7 centric chromosome fusions, respectively.

The second, “eastern and coastal” clade, includes 11 karyotyped representatives. Five species – *E.grahami*, *E.infrafasciatus*, *E.sangmelinensis*, *E.mesogramma* and *E.chevalieri* – share cytogenetic characteristics close to the putative ancestral state, occupying different positions on the phylogenetic tree. In *E.esekanus* (2n = 42; FN = 50), *E.huberi* (2n = 46, FN = 52) and *E.singa* (2n = 42) chromosome number is independently reduced presumably by centric fusions, similar to members of the first clade. The closely related *E.chaperi*, *E.dageti* and *E.annulatus* have 2n = 50. It can be suggested that these karyotypes were derived by tandem fissions. It is noteworthy that in *E.annulatus* (FN = 70) and *E.dageti* (FN = 82/86) the number of chromosome arms is greatly increased, probably due to pericentric inversions or other types of centromere repositioning.

Among the karyotyped species for which the position on the phylogenetic tree is not known, it is important to mention *E.ansorgii* (2n = 46/48, FN = 50), *E.barmoiensis* (2n = 34, FN = 48), *E.multifasciatus* (2n = 46, FN = 60) and *E.olbrechtsi* (2n = 38, FN = 50), in which 2n is reduced probably due to centric chromosome fusions, similar to the representatives of the first and second clades. The karyotype of *E.multifasciatus* also has a markedly increased number of biarmed elements. *E.sexfasciatus* (2n = 48, FN = 48), a species also of an unknown phylogenetic position, has cytogenetic characteristics close to the putative ancestral state.

To summarize, chromosome evolution in the genus *Epiplatys* is characterized by the following: 1) the putative ancestral karyotype with 2n = 48 and FN = 48; 2) independent events of structural rearrangements of centric fusion type; 3) increase in the number of chromosomal arms by pericentric inversions or other types of centromere repositioning, and 4) at least one chromosome fission event. The cytogenetic study in the genus *Epiplatys* is interesting in the context of the study of the family Nothobranchiidae, which is characterized by high rates of chromosome and genome evolution, with maximum expression in African annual killifishes of the genus *Nothobranchius* Peters, 1868 (Krysanov, Demidova 2018; Cui et al. 2019; Štundlová et al. 2022; Krysanov et al. 2023; Lukšíková et al. 2023; Voleníková et al. 2023). Based on the phylogenetic tree (Cui et al. 2019), it can be assumed that the genus *Epiplatys* shares more basal features of karyotype differentiation compared to the genus *Nothobranchius*.
